# *Candida albicans* Yeast and Hyphae are Discriminated by MAPK Signaling in Vaginal Epithelial Cells

**DOI:** 10.1371/journal.pone.0026580

**Published:** 2011-11-08

**Authors:** David L. Moyes, Celia Murciano, Manohursingh Runglall, Ayesha Islam, Selvam Thavaraj, Julian R. Naglik

**Affiliations:** Department of Oral Medicine, Pathology and Immunology, King's College London Dental Institute, King's College London, London, United Kingdom; University of Aberdeen, United Kingdom

## Abstract

We previously reported that a bi-phasic innate immune MAPK response, constituting activation of the mitogen-activated protein kinase (MAPK) phosphatase MKP1 and c-Fos transcription factor, discriminates between the yeast and hyphal forms of *Candida albicans* in oral epithelial cells (ECs). Since the vast majority of mucosal *Candida* infections are vaginal, we sought to determine whether a similar bi-phasic MAPK-based immune response was activated by *C. albicans* in vaginal ECs. Here, we demonstrate that vaginal ECs orchestrate an innate response to *C. albicans* via NF-κB and MAPK signaling pathways. However, unlike in oral ECs, the first MAPK response, defined by c-Jun transcription factor activation, is delayed until 2 h in vaginal ECs but is still independent of hypha formation. The ‘second’ or ‘late’ MAPK response, constituting MKP1 and c-Fos transcription factor activation, is identical to oral ECs and is dependent upon both hypha formation and fungal burdens. NF-κB activation is immediate but independent of morphology. Furthermore, the proinflammatory response in vaginal ECs is different to oral ECs, with an absence of G-CSF and CCL20 and low level IL-6 production. Therefore, differences exist in how *C. albicans* activates signaling mechanisms in oral and vaginal ECs; however, the activation of MAPK-based pathways that discriminate between yeast and hyphal forms is retained between these mucosal sites. We conclude that this MAPK-based signaling pathway is a common mechanism enabling different human epithelial tissues to orchestrate innate immune responses specifically against *C. albicans* hyphae.

## Introduction

The mucosal epithelium is of immense importance in host defense and immune surveillance, as it is the initial tissue encountered by the majority of infecting microorganisms. Vaginal epithelium provides a physical barrier, which recognizes commensal and pathogenic microbes, as well as regulating the influx of immune cells to prevent inflammatory tissue destruction. This specialized interaction between microbes, epithelial cells (ECs) and local immune cells results in either a degree of mutualism between microbe and host, as in the case of commensal microbes, or a breach of the mucosal barrier and subsequent cell injury, as in the case of pathogenic microbes. Indeed, the integrity of the host immune system plays an important role in defining whether a microbe acts as a commensal colonizer or as an opportunistic pathogen. Of particular interest are ‘opportunistic’ microbes, which although normally commensal are capable of becoming pathogenic. The polymorphic fungus *Candida albicans* is one such opportunistic microbe, being a constituent of the normal vaginal microbiota but commonly causing mucosal disease in healthy women of fertile age [1].

Recently, we identified a host mechanism in oral ECs that discriminates between the commensal and pathogenic states of *C. albicans*, which is based on hypha recognition and fungal burdens [2]. We demonstrated that epithelial innate immunity against *C. albicans* is initiated via NF-κB and a bi-phasic mitogen-activated protein kinase (MAPK) response. Activation of NF-κB and the first MAPK phase, constituting activation of the c-Jun transcription factor, is independent of morphology and is due to the recognition of fungal cell wall polysaccharides (chitin, mannan, β-glucan). Activation of the second MAPK phase, constituting phosphorylation of the MAPK phosphatase MKP1 and activation of the c-Fos transcription factor, is specifically induced by *C. albicans* hyphae and correlates with proinflammatory responses and cell damage in a dose-dependent manner. This proinflammatory response is thought to recruit neutrophils and protects against oral fungal infection via a novel mechanism involving epithelial toll-like receptor (TLR) 4 [3]. However, neutrophil recruitment at the vaginal mucosa is thought to be detrimental to the host [4] and does not necessarily result in clearance of the fungal infection, demonstrating fundamental immunological differences in responses to *C. albicans* at these two mucosal sites.

Given our findings in oral ECs, in this study we sought to determine how *C. albicans* activates vaginal ECs and whether a similar MAPK/MKP1/c-Fos discriminatory system also exists, with the aim of potentially identifying a common mechanism enabling different epithelial tissues to identify when this normally commensal fungus switches to hyphal growth associated with invasion and pathology.

## Materials and Methods

### Cell lines, reagents and *Candida albicans* strains

Experiments were carried out using the A431 human vulval epidermoid carcinoma cell line and the TR146 buccal epithelial carcinoma cell line (SkinEthic Laboratories, Nice, France). Monolayer epithelial cultures were grown in Dulbecco's Modified Eagle's Medium (DMEM) (PAA, UK) supplemented with 10% fetal bovine serum (FBS) (PAA, UK) and experiments carried out in serum-free DMEM. Reconstituted human vaginal epithelium (vaginal RHE: 5-day) were purchased from SkinEthic Laboratories (Nice, France) and used as previously described [2], [5]. This model is created from the same cell line (A431) and constitutes several layers of stratified squamous epithelium permitting the direct analysis of pathogen-epithelial interactions that are not complicated by non-epithelial factors. Antibodies to phospho-p38, phospho-ERK1/2, phospho-JNK, phospho-MKP1, phospho-IκBα, IκBα and c-Fos were purchased from Cell Signalling Technologies (New England Biolabs, UK). Mouse monoclonal antibody to human α-actin was purchased from Chemicon (Millipore) and goat anti-mouse and anti-rabbit horseradish peroxidase (HRP)-conjugated antibodies were purchased from Jackson Immunologicals Ltd (Stratech Scientific, UK). Fungal strains included *C. albicans* wild-type SC5314 [6], CAI4 (+CIp10) (NGY152) [7], Δ*nrg1* (MMC4) [8] and Δ*efg1/cph1* (Can36) [9]. All strains were grown in YPD medium (1% Yeast Extract, 2% Peptone, 2% Dextrose) overnight at 30°C to stationary phase prior to experimentation.

### Fungal infection of epithelium

*C. albicans* strains were inoculated at 2×10^6^ cells after being washed twice in sterile PBS onto vaginal RHE, or between10^4–7^ cells/ml for signaling assays and cytokine assays on monolayer epithelial cultures. The multiplicity of infection (MOI) ranged from 0.01–10 (fungal cells per EC) depending on the experiment. Vaginal RHE and monolayers were incubated at 37°C in 5% CO_2_ for 5, 15, 30, 60 min, 2, 3 or 24 h as previously described [2], [5], [10] depending on the experiment. Non-infected controls contained PBS alone.

### Western blotting

ECs were lysed using a modified RIPA lysis buffer (50 mM Tris-HCl (pH 7.4), 150 mM NaCl, 1 mM EDTA, 1% Triton x-100, 1% Sodium deoxycholate, 0.1% SDS) containing protease (Sigma-Aldrich) and phosphatase inhibitors (Perbio), left on ice for 30 min, and then centrifuged for 10 min in a refrigerated microfuge. Supernatants were assayed for total protein using the BCA protein quantitation kit (Perbio). 20 µg of protein was separated on 12% SDS-PAGE gels before transfer to PVDF membranes (GE Healthcare, UK). After probing with primary and secondary antibodies (dilutions vary from 1∶1000 to 1∶10,000), membranes were developed using Immobilon chemiluminescent substrate (Millipore, UK) and exposed to ECL film (GE Healthcare, UK). α-actin was used as a loading control.

### Transcription factor DNA binding assay

c-Fos DNA binding activity was assessed using the TransAM transcription factor ELISA system (Active Motif, Belgium). Briefly, nuclear proteins were isolated from ECs after 3 h infection with *C. albicans* using a nuclear protein extraction kit as per manufacturer's instructions (Active Motif, Belgium). Protein concentration was determined as above and 5 µg of nuclear extract was assayed in the TransAM system according to the manufacturer's protocol.

### Cytokine determination

Cytokine levels (IL-1α, IL-6, IL-8, G-CSF, GM-CSF, TNFα, MCP-1 (CCL2) and MIP-1α (CCL3)) in cell culture supernatants were determined at 24 h using the Fluorokine MAP cytokine multiplex kits (R&D Systems), coupled with the Luminex 100™ machine according to the manufacturer's protocol. The trimmed median value was used to derive the standard curve and calculate sample concentrations. Standard curve ranges were as follows: IL-1α 1,850 pg/ml – 2.5 pg/ml; IL-6 4,950 pg/ml – 6.8 pg/ml; IL-8 2,950 pg/ml – 4 pg/ml; G-CSF 4,500 pg/ml – 6.2 pg/ml; GM-CSF 2,850 – 3.9 pg/ml. CCL20 (MIP-3α) was analyzed using the Duoset ELISA kit (R&D Systems) and standard curve range was 1,000 pg/ml – 15.6 pg/ml. Values for both assays were only regarded as valid if they fell within the standard curve range.

### Epithelial cell damage assay

EC damage was determined at 24 h by measuring lactate dehydrogenase (LDH) activity in the culture supernatant as described previously [2], [5], [10]. This was performed using the Cytox 96 Non-Radioactive Cytotoxicity Assay kit (Promega) according to the manufacturer's protocol and using a recombinant porcine LDH (Sigma-Aldrich) to generate a standard curve. Sample values were then extrapolated from this curve.

### Immunohistochemistry of vaginal RHE

*C. albicans* infected vaginal RHE was fixed in 10% (v/v) formal-saline before being embedded and processed in paraffin wax using standard protocol. 5 µm sections were prepared using a Leica RM2055 microtome and silane coated slides. After dewaxing in xylene, protein expression was determined using rabbit anti-human polyclonal antibodies for MKP1 (Santa Cruz Biotechnology) and c-Fos (Source Bioscience) (1∶10 and 1∶100, respectively) and counterstained with peroxidase-conjugated goat anti-rabbit secondary IgG antibody, followed by diaminobenzidine (DAB) chromogen detection as per manufacturer's protocol. To visualize *C. albicans*, sections were stained using Periodic Acid Schiff (PAS), counterstained with haematoxylin and examined by light microscopy.

### Statistics

Data were analyzed in the GraphPad software package using either the unpaired two tailed t-test or in the case of hyphae vs. yeast cytokine comparisons, ANOVA with Bonferroni post-hoc analysis. In all cases, p<0.05 was taken to be significant.

## Results

### Activation of the NF-κB and MAPK signaling pathways

**Figure 1:**
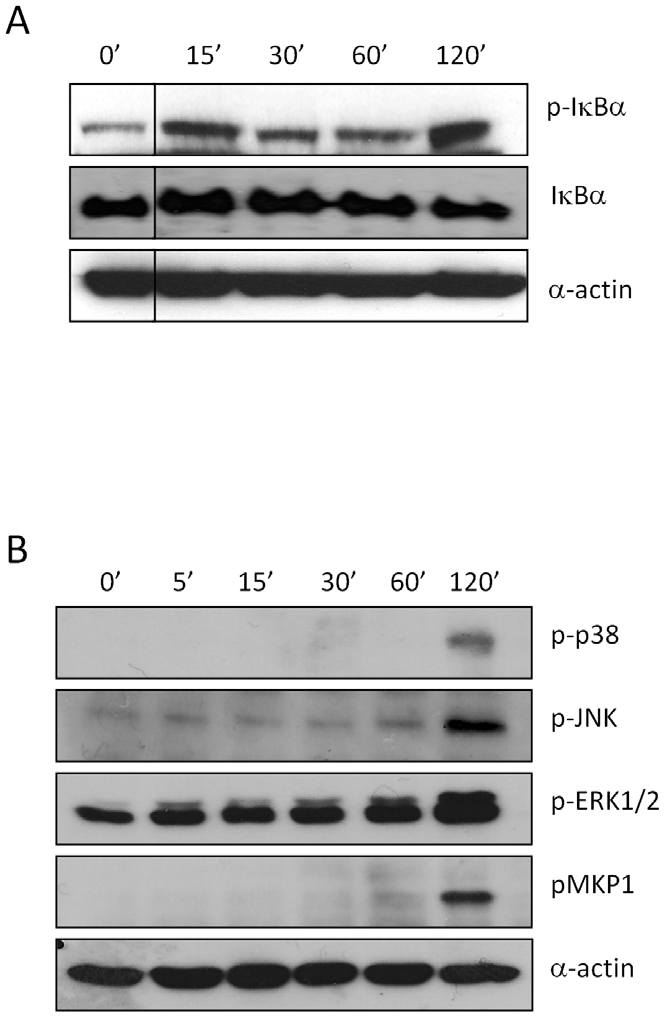
*C. albicans* infection of A431 epithelial cells activates NF-κB and MAPK signaling. *C. albicans* SC5314 was added to A431 vaginal ECs under standard culture conditions for 5, 15, 30, 60 min and 2 h. Total protein was isolated and phosphorylation of (A) IκBα or (B) p38, JNK, ERK1/2 and MKP1 were assessed by Western blotting. Bands are shown with an α-actin loading control. A *C. albicans*:EC MOI of 10∶1 was used. Data are representative of three independent experiments.

A431 vaginal ECs were infected with *C. albicans* SC5314 and IκB-α phosphorylation, which is a key event in NF-κB pathway activation, and ERK1/2, JNK and p38 phosphorylation (MAPK activation) was determined after 5, 15, 30, 60 and 120 min (5 min not performed for IκB-α). Immunoblot analysis confirmed that IκB-α phosphorylation occurs immediately, being detectable 15 min post-infection, and persists over a 2 h period (Fig. 1A). Interestingly, there was a lack of JNK and p38 phosphorylation and only a slight increase in the phosphorylation of ERK1/2 at early time points (5–60 min) but strong activation of all three MAPK proteins at 2 h post-infection (Fig. 1B). We next explored downstream MAPK-induced events by investigating MKP1 (DUSP1) stabilization. MKP1 is a key phosphatase involved in a negative feedback loop regulating the MAPK pathway [11] and is phosphorylated by ERK1/2 to prevent its degradation. This event is independent of gene transcription and is mediated by ERK1/2 [12]. Phosphorylation of MKP1 only appeared at 2 h post-infection (Fig. 1B), coinciding with phosphorylation of the three MAPK proteins. In summary, in vaginal ECs the early MAPK response against *C. albicans* appears to be absent but the late MAPK response is activated culminating in stabilization of MKP1.

### Activation of MAPK transcription factors

**Figure 2:**
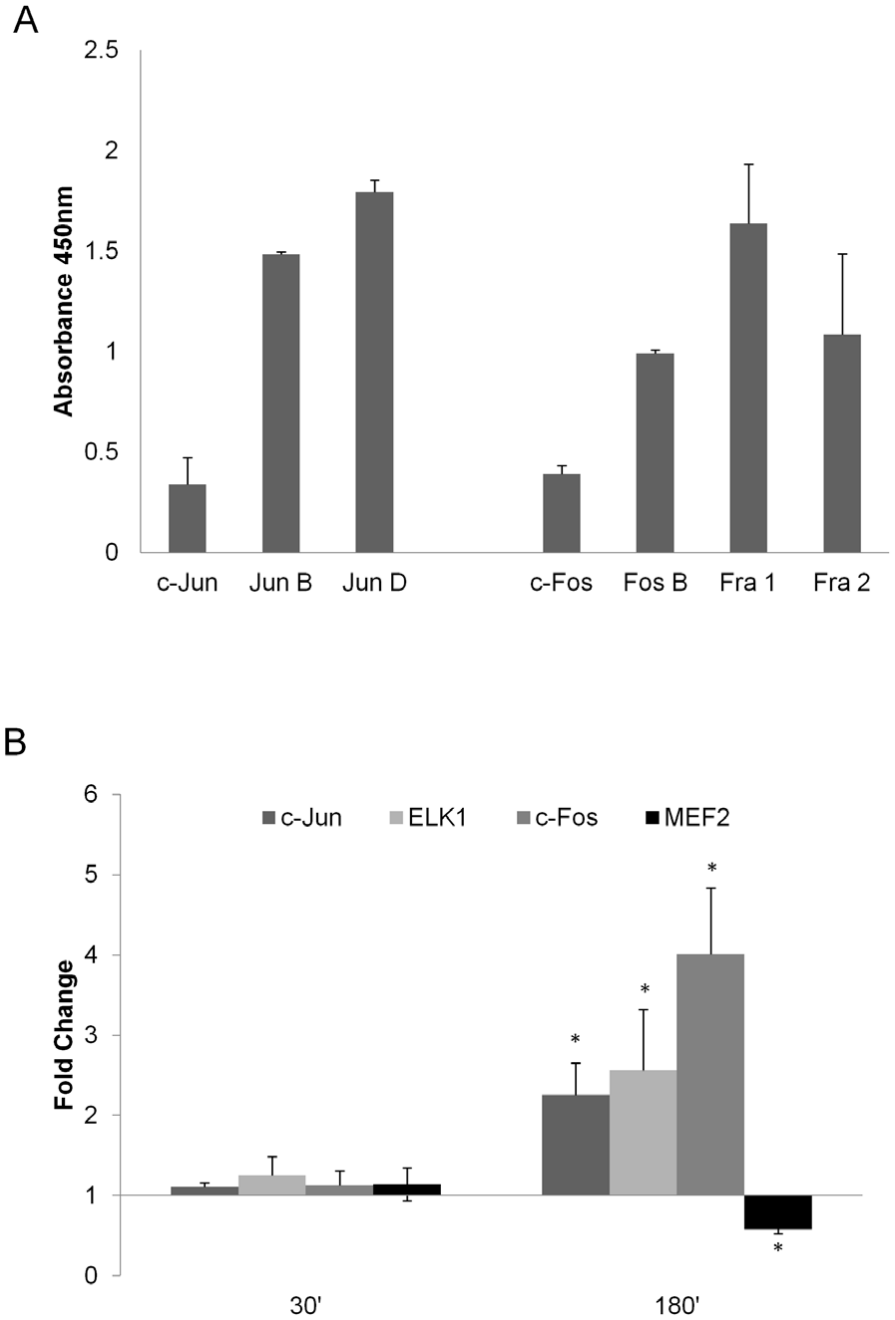
Activation of MAPK transcription factors by *C. albicans* in A431 vaginal epithelial cells. (A) Resting levels of DNA binding activity (absorbance values) of AP-1 transcription factor members in nuclear extracts from A431 ECs, measured by TransAm ELISA. (B) Changes in DNA binding activity of c-Jun, Elk1, c-Fos, and MEF2 in nuclear extracts of A431 ECs 30 and 180 min post-infection with *C. albicans* SC5314 by TransAm ELISA. These MAPK-activated transcription factors have previously been identified as showing altered DNA binding activity in oral ECs. Data are represented as fold change relative to resting levels at 0 h. A *C. albicans*:EC MOI of 10∶1 was used. Data represent mean values ± SEM and are representative of a minimum of three independent experiments. Statistical analysis (B) of raw data for infected versus uninfected cells was performed using the unpaired, two-tailed t-test * p<0.05.

MAPK signaling is associated with activation of the AP-1 transcription factor complex, which is a heterodimer of a Jun family member (JunB, JunD, c-Jun) and a Fos family member (Fra1, Fra2, c-Fos, and FosB) [13], [14]. Given the temporal differences we observed in MAPK activation between vaginal ECs (Fig. 1B) and oral ECs [2], we determined changes in DNA binding activity (and thus activation) of all AP-1 family members 30 min and 3 h post-infection with *C. albicans*, which were previously determined as optimal time points in oral ECs to measure DNA binding activity [2]. In resting vaginal ECs, DNA binding activity is present for all members of the Jun and Fos families (Fig. 2A). Interestingly, whilst no changes in AP-1 transcription factor binding was observed at 30 min post-infection (early response), by 3 h both c-Jun and c-Fos binding was significantly increased compared with the PBS control (p<0.05) (Fig. 2B). This activity at the transcription factor level (c-Jun and c-Fos) parallels activation of the MAPK response at the signal pathway level (Fig. 1B). The binding activity of other Jun or Fos proteins at 3 h was unaltered (data not shown). As well as the AP-1 family, we investigated the binding activity of other MAPK-induced transcription factors, including c-Myc, ATF2, Elk-1, and MEF2. Elk-1 DNA binding activity mirrored c-Jun DNA binding activity in that there was no change at 30 min but was increased at 3 h (p<0.05) (Fig. 2B). In contrast, MEF2 DNA binding activity also showed no change at 30 min but was decreased at 3 h (p<0.05) (Fig. 2B). DNA binding activity of ATF2 and c-Myc was unchanged at both time points (data not shown). The data demonstrate that vaginal ECs direct a specific and organized response to *C. albicans*, whereby changes in transcription factor binding activity occurred at 3 h post-infection, with distinct profiles of activity that either mirror (c-Fos increased and MEF2 decreased) or differ (c-Jun increased at 3 h, Elk-1 increased at 3 h, not decreased at 2 h) from those seen in oral ECs.

### Vaginal and oral cytokine profiles differ

**Figure 3:**
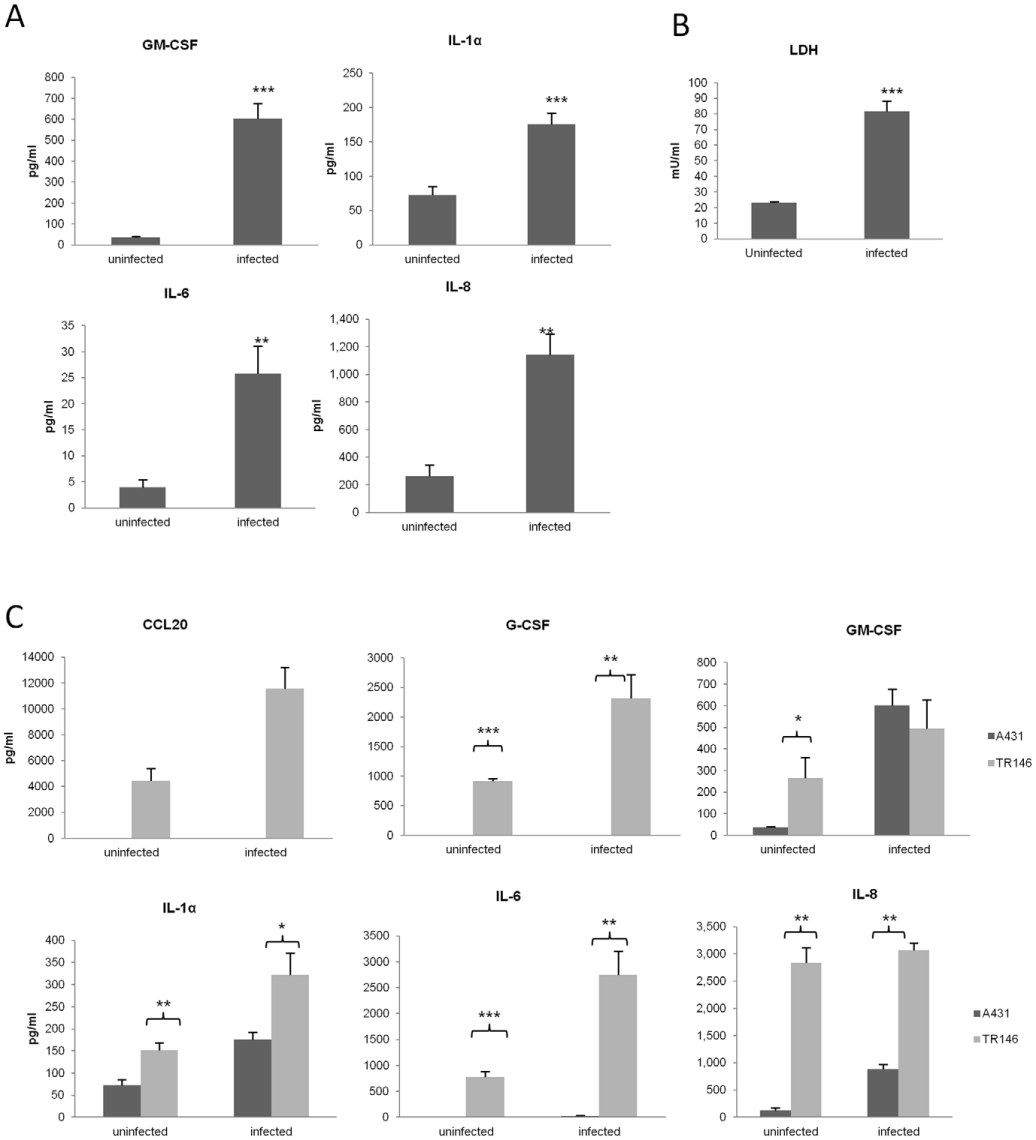
Cytokine activation and cell damage induced by *C. albicans* in A431 vaginal epithelial cells and TR146 oral epithelial cells. (A) *C. albicans* SC5314 was added to monolayers of A431 ECs for 24 h and the cell culture medium collected and assessed for cytokine proteins by multiplex microbead assay (luminex) or ELISA (CCL20). (B) LDH (lactate dehydrogenase) release (measure of cell damage) from A431 ECs 24 h post-infection with *C. albicans* SC5314. (C) Comparison of cytokine protein release by A431 ECs and TR146 ECs 24 h post-infection with *C. albicans* SC5314. A *C. albicans*:EC MOI of 0.01 was used for all the experiments. Data are mean values ± SEM of three independent experiments. Statistical analysis of infected versus uninfected epithelial cells (A & B) or oral versus vaginal epithelial cells (C) was performed using the unpaired, two-tailed t-test. * p<0.05, ** p<0.01, *** p<0.001.

MKP1 and c-Fos activation correlates with cytokine production in response to *C. albicans* infection in oral ECs [2]. Therefore, we hypothesized that *C. albicans* infection would also induce cytokine production in vaginal ECs. A431 monolayers were infected with *C. albicans* at an MOI of 0.01 (this having previously been determined as the optimal ratio of fungal cells for a 24 h infection study [2]. Analysis of culture supernatants of A431 epithelial monolayers 24 h post-infection with *C. albicans* SC5314 demonstrated that of the nine cytokines and chemokines assessed (IL-1α, IL-6, IL-8, G-CSF, GM-CSF, TNFα, MCP-1 (CCL2), MIP-1α (CCL3) and MIP-3α (CCL20)), only three were significantly induced by *C. albicans*: IL-1α, IL-8 and GM-CSF, with IL-6 induced in very low amounts, although still showing a 4-fold increase over resting cells (Fig. 3A). As with oral ECs, *C. albicans* induces significant damage to vaginal ECs after 24 h as measured by LDH release (Fig. 3B) with a concurrent release of the damage-associated cytokine IL-1α (Fig. 3A). Since *C. albicans* infection of oral ECs induces additional cytokines including G-CSF and CCL20 [2], [15]), we re-assessed the oral TR146 ECs cytokine response and compared this with the A431 vaginal ECs cytokine response. Fig. 3C confirms the absence of CCL20, very low level of G-CSF, and low level IL-6 secretion by A431 vaginal ECs as compared with TR146 oral ECs.

### c-Fos activation and cytokine production is dependent on *C. albicans* morphology

**Figure 4:**
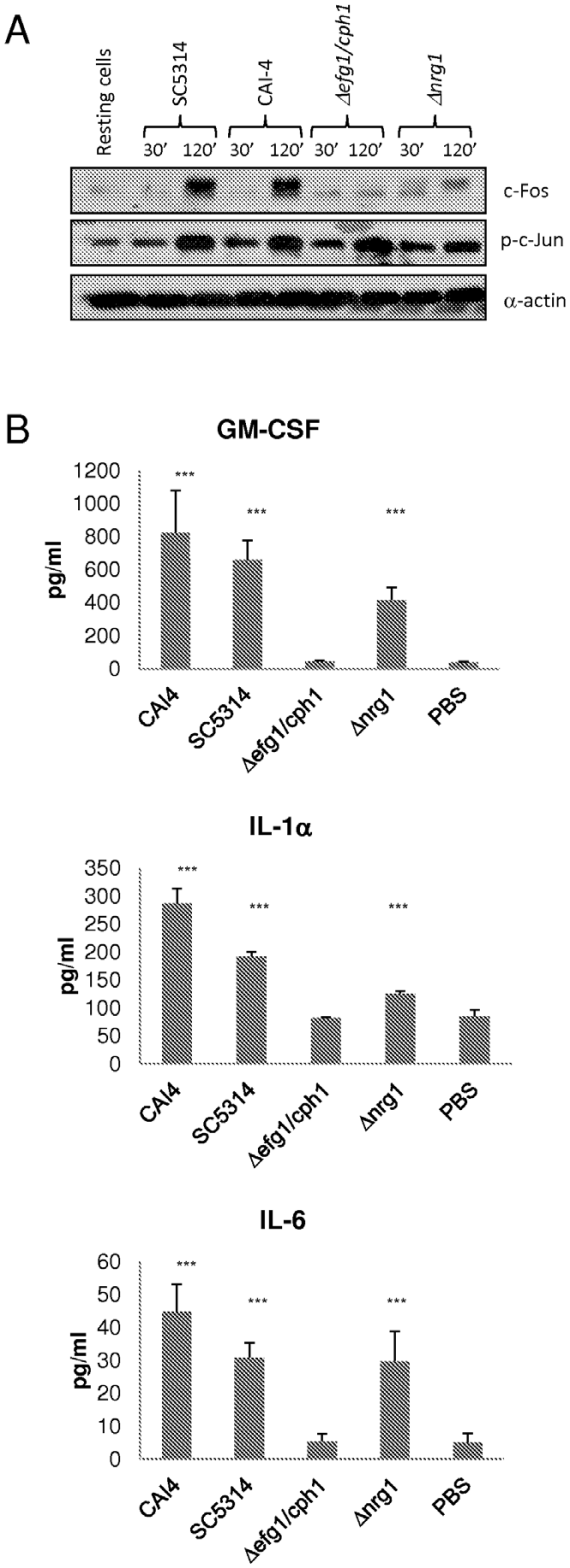
Induction of c-Jun phosphorylation, c-Fos and cytokine production in A431 vaginal epithelial cells is dependent on *C. albicans* hypha formation. (A) *C. albicans* SC5314 (wild type), CAI4 (parent control), Δ*efg1/cph1* (non-filamentous) and Δ*nrg1* (hyperfilamentous) were added to A431 vaginal ECs under standard culture conditions for 30 min and 2 h. Total protein was isolated and induction of c-Jun phosphorylation and c-Fos assessed by Western blotting. Bands are shown alongside an α-actin loading control. (B) Cytokine protein production by A431 ECs 24 h post-infection with all four strains as measured by multiplex microbead assay (luminex). A *C. albicans*:EC MOI of 10∶1 (A) and 0.01 (B) was used. Data are representative (A) or mean ± SEM (B) of three independent experiments. Statistical analysis (B) of SC5314, CAI-4, Δ*efg1/cph1* and Δ*nrg1* infected versus PBS-treated controls was performed using the ANOVA test with Bonferroni post-hoc analysis. *** = p<0.001.

*C. albicans* infection of vaginal ECs results in yeast-to-hyphal transition by 1 h. Therefore, we hypothesized that epithelial activation might be due to a specific response to hypha formation, just like in oral ECs [2]. To test this, we infected A431 cells with *C. albicans* SC5314 and two mutants strains,Δ*efg1/cph1* (unable to form hyphae) and Δ*nrg1* (hyperfilamentous), for 30 min and 2 h and tested for induction of the two transcription factors associated with the bi-phasic MAPK-based response in oral ECs: c-Fos (specific response to hyphae) and c-Jun (general response to *C. albicans*). Furthermore, because in vaginal ECs c-Jun phosphorylation was part of the late (2 h) MAPK-mediated response (Fig. 1B) (when hypha formation is evident), we wanted to determine whether c-Jun phosphorylation was hypha-dependent or just a delayed response to the presence of *C. albicans* (and thus hypha-independent). The respective morphological phenotypes of these mutants were maintained throughout all experiments.

c-Fos was not induced at either time point by the Δ*efg1/cph1* non-filamentous mutant but was induced at 2 h by the Δ*nrg1* hyperfilamentous mutant, albeit less than the wild-type *C. albicans* SC5314 (Fig. 4A), confirming that c-Fos induction in vaginal ECs is also dependent on hypha formation. The difference between Δ*nrg1* and SC5314 can be explained partly by the reduced level of adherence of Δ*nrg1* as compared with SC5314 and partly by the lack of fungal-EC contact i.e. the SC5314 cells settled rapidly onto the epithelial surface prior to hypha formation whereas the Δ*nrg1* hyperfilamentous strain ‘floated’ for a prolonged period of time before gradually settling, thus reducing the MOI and threshold level of activation [2]. Notably, all three *C. albicans* strains phosphorylated c-Jun at 2 h but not at 30 min (Fig. 4A), demonstrating that c-Jun phosphorylation appears to be independent of hypha formation i.e. is a delayed general response to *C. albicans*. Given the correlation between c-Fos induction and cytokine production we wanted to confirm that the hypha deficient strain was unable to induce cytokines since it was unable to induce c-Fos. Figure 4B confirms that the non-filamentous strain (Δ*efg1/cph1*) failed to induce cytokine production, whereas the hyperfilamentous strain (Δ*nrg1*) was able to induce IL-1α, IL-6 and GM-CSF albeit to a lesser extent than the wild type (SC5314), probably for the reasons stated above. Since Δ*efg1/cph1* was able to induce c-Jun activation (Fig. 4A) but was unable to induce cytokines (Fig. 4B), this suggests that c-Jun does not contribute to cytokine production. Together, the data indicate that in vaginal ECs c-Fos activation is hypha specific and is required for cytokine production, whereas c-Jun activation is delayed, independent of hypha formation and does not contribute to cytokine induction.

### MAPK/MKP1/c-Fos response is dependent on fungal burden

**Figure 5:**
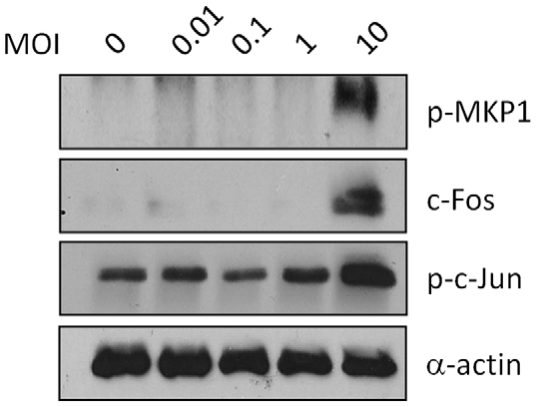
Effect of fungal burdens on MKP1, c-Fos and c-Jun activation. A431 vaginal ECs were infected for 2 h with *C. albicans* SC5314 at MOI's ranging between 0.01 and 10. Total protein was isolated and phosphorylation of MKP1 and c-Jun and c-Fos induction was assessed by Western blotting. Bands are shown alongside an α-actin loading control. Data are representative of four independent experiments.

Activation of the MAPK/MKP1/c-Fos response in oral ECs is dependent on fungal burden, which may represent a danger response that is only activated if sufficient *C. albicans* hyphae are present [2]. To determine whether a similar threshold level of activation is required in vaginal ECs, we stimulated A431 cells with different doses of *C. albicans* SC5314 (10^4^–10^7^ cells/ml). This was equivalent to an MOI of 0.01–10 *C. albicans* cells per EC. Interestingly, induction of MKP1 and c-Jun phosphorylation and c-Fos were observed only at the highest MOI of 10 (Fig. 5), which is a log greater than oral ECs [2].

### MKP1 and c-Fos activation in an RHE model of vaginal epithelium

**Figure 6:**
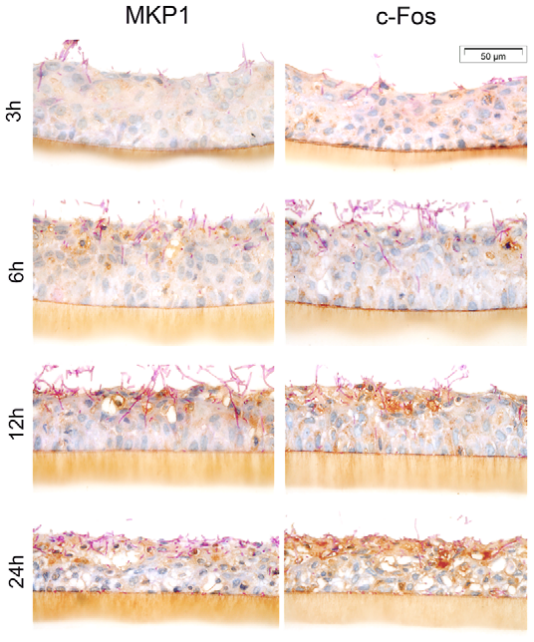
Expression of MKP1 and c-Fos in vaginal RHE. Increase in MKP1 and c-Fos expression in vaginal epithelium is associated with *C. albicans* SC5314 hypha formation, with minimal activation at the surface by 3 h and gradually increased activation co-localising in the epithelial layers where hyphae penetrate and invade at 6, 12 and 24 h (dark brown staining). Resting levels of MKP1 and c-Fos expression can be seen in areas without *C. albicans* at each time point (sectional control).

Finally, to obtain more *in vivo* relevant data, we determined the presence of MKP1 and c-Fos expression in RHE models of human vaginal epithelium after 3, 6, 12 and 24 h post-infection with *C. albicans* SC5314. c-Jun was not investigated as c-Jun activation was not hypha-dependent or associated with epithelial activation (see data above). Figure 6 shows a gradual intensification of MKP1 and c-Fos expression (brown staining) as time proceeds from 3–24 h post-infection. Expression can be observed in regions of hyphal contact moving from the surface at early time points (3 h) to deeper epithelial layers at later time points (24 h). A clear delineation can be observed with cells in contact with hyphae showing expression of both MKP1 and c-Fos, whilst cells in adjacent uninfected areas show little increase in MKP1 and c-Fos expression.

## Discussion

Discrimination between the yeast and hyphal form of *C. albicans* appears to be a crucial event for epithelial immune activation and fungal pathogenicity [2], [5], [16], [17]. Previously, we demonstrated that oral ECs initiate innate immunity against *C. albicans* via a bi-phasic MAPK response. MAPK activation is key to hypha discrimination and constitutes phosphorylation of the MAPK phosphatase MKP1, activation of the c-Fos transcription factor, and induction of a proinflammatory response [2]. Here, we report that differences exist in how vaginal ECs initially respond to *C. albicans* as compared with oral ECs, but that a near-identical MAPK-based mechanism discriminates between the yeast and hyphal form of *C. albicans*. We propose that this MAPK/MKP1/c-Fos-based signaling system identifies a common mechanism playing a central role in enabling different human epithelial tissues to recognize *C. albicans* hyphae and initiate innate immune responses.

We found that NF-κB responses in vaginal ECs are the same as for oral ECs, indicating that this would appear to be a generic EC response to *C. albicans*. In contrast, the pattern of MAPK activation in vaginal ECs differs from oral ECs. Although the hyphal discrimination response (c-Fos and MEF2 DNA binding activity and MKP1 phosphorylation) is identical in both cell types, there are major differences in the generic *Candida* recognition response (c-Jun activation/phosphorylation). Unlike oral ECs, vaginal ECs show a delayed response (2 h instead of 30 min). As well as the delay in activation, Elk-1 also shows an increase rather than the decrease in activity seen in oral ECs. This data suggested one of two possibilities. Either the early MAPK response to the yeast form is ‘delayed’ but still occurs at later time points coinciding with the hyphal-mediated activation of MKP1 and c-Fos, or vaginal ECs are unresponsive to the yeast form via the MAPK pathways and respond only to the hyphal form to activate both c-Jun and c-Fos. However, the *C. albicans* hyphal deficient strain (Δ*efg1/cph1*) was able to induce c-Jun phosphorylation but not c-Fos at 3 h whilst the hyperfilamentous strain (Δ*nrg1*) induced both c-Jun and c-Fos. This indicates that c-Fos activation in vaginal ECs is hypha specific, whereas c-Jun activation is a delayed response to *C. albicans* yeast and independent of hypha formation. We then assessed induction of the hypha-associated response by analysing MKP1 and c-Fos expression in an RHE model of vaginal infection and found a gradual intensification of MKP1 and c-Fos expression as *C. albicans* infection progressed. This confirmed the association of MKP1 and c-Fos induction with hypha formation. From these data we conclude that (i) initial recognition of *C. albicans* yeasts in vaginal ECs is mediated solely by the NF-κB pathway, whereas in oral ECs it is mediated by both NF-κB and MAPK pathways, and (ii) both c-Jun and c-Fos transcription factors are activated in vaginal ECs in the late MAPK response to *C. albicans* whereas only c-Fos is activated in oral ECs, but in both EC types only c-Fos activation is hypha specific.

Of major importance is the finding that this MAPK/MKP1/c-Fos response mechanism is dependent not only on hypha formation but also on fungal burdens and suggests that a threshold level of stimulation is required prior to full activation of the epithelial innate response. This may provide a mechanism by which epithelial tissues can remain quiescent in the presence of low fungal burdens (even if hyphae are present) whilst responding specifically and strongly to damage-inducing hyphae as burdens increase. However, of particular interest was the finding that induction of MKP1 and c-Jun phosphorylation and c-Fos was observed only at the highest MOI of 10, one log greater than in oral ECs [2]. This indicates that the responsiveness of vaginal ECs to *C. albicans* is lower than that of oral ECs and that vaginal ECs may be able to tolerate greater fungal (hyphal) burdens before epithelial activation is initiated. The importance of this difference in fungal burdens to immunity at these two surfaces can be seen more clearly when responses *in vivo* to candidiasis is considered. In oral mucosa, neutrophils play an important role in clearing or combating infections by *C. albicans*. However, the situation in vaginal mucosa may be different, where neutropenia does not have a major impact on *Candida* burdens but does result in reduced inflammation [18], [19]. Importantly, vaginal ECs have a direct fungistatic effect that does not require EC viability, thus controlling the burden of *C. albicans* without recourse to neutrophils or other immune cells [20]–[23]. With the discovery that women with infrequent or recurrent vulvovaginal candidiasis show symptomatic disease at correspondingly lower *Candida* burdens [4] and that this increased sensitivity is due to EC responsiveness, we can hypothesize that vaginal ECs play a key role in managing *Candida* burdens at vaginal mucosal surfaces, controlling fungal burden in a passive manner but driving pathological inflammation when they become activated. This inflammation is driven by the recruitment of neutrophils by ECs, which may result in uncontrolled inflammation [24].

Although the initial recognition of *C. albicans* yeast cells appears to be via NF-κB, this does not necessarily result in immunostimulation as the hyphal deficient strain Δ*efg1/cph1* was unable to induce cytokines after 24 h despite activating NF-κB. This lack of cytokine induction by *C. albicans* yeast cells is common to oral ECs [2] but is in contrast to myeloid/lymphoid cells where strong cytokine responses are induced by yeast cells [25]–[27]. Similar effects have been reported in gut ECs, where both NF-κB and p38 signaling are required for full activation of inflammation in the gut [28]. In contrast, wild-type *C. albicans* and the hyperfilamentous strain Δ*nrg1* induced cytokine secretion (IL-1α, GM-CSF and IL-6) in vaginal ECs, which correlated with induction of MAPK pathways, MKP1 phosphorylation and c-Fos activation. Our combined data indicate that ECs from different mucosal sites (oral and vaginal) respond to *C. albicans* differently to that of myeloid/lymphoid cells by specifically targeting the hyphal form of the fungus, which leads to differential cytokine production. We note that the shift in morphology from yeast to hyphae results in the expression of many different potential virulence factors, such as secreted aspartyl proteases (Saps) and adhesins and it may be that the lack of vaginal EC responses to the hyphal deficient strain Δ*efg1/cph1* is partly due to the lack of production of such virulence factors. The identities of the hyphal moieties or surface EC receptors that induce/mediate vaginal epithelial activation are currently unknown but will be the focus of future studies.

Cytokine induction was dependent upon hypha formation correlating with c-Fos activation, MKP1 stabilization and cell damage. Like in oral ECs, we propose that activation of this MAPK-based response represents a ‘danger response’ mechanism informing the host of invading hyphae. Interestingly, the cytokines secreted by vaginal ECs in response to *C. albicans* differed to the cytokines secreted by oral ECs, despite the same signaling pathways being activated (NF-κB and MAPK). We and others have shown that oral EC's secrete IL-1α, IL-6, GM-CSF, G-CSF, IL-8 and CCL20 [2], [3], [29], [30], whereas vaginal ECs only secreted IL-1α, IL-8 and GM-CSF (this study: IL-6 is released but at very low levels compared with oral ECs). This suggests that although a common signaling recognition system is utilized by both EC lineages to detect *C. albicans*, downstream induction of immune effector responses can differ, demonstrating that a further level of immunoregulation probably exists at latter stages of EC activation. Given the established link between IL-1α and cell damage [31], [32], the secretion of IL-1α by both oral and vaginal ECs is probably the result of hypha-induced cell damage. IL-8 and GM-CSF secretion by both EC types will function to recruit and activate neutrophils to the site of mucosal infection, which is a well established phenomenon [33].

The potential importance *in vivo* of why IL-6, G-CSF and CCL20 are selectively induced by *C. albicans* in oral ECs but not vaginal ECs is not known. IL-6 can act as both a pro-inflammatory and anti-inflammatory cytokine and G-CSF stimulates the proliferation, differentiation and function of neutrophils. Neutrophil recruitment to vaginal tissues during candidiasis occurs in humans [4] and mice [24]. However, in humans, neutrophil recruitment appears to have a detrimental effect, resulting in acute inflammation and thus symptoms associated with vaginitis [4]. In contrast, recruitment of neutrophils in human oral *Candida* infection is regarded as beneficial [34], [35] and has been shown to protect against infection in an RHE model of oral candidiasis [3]. In addition, neutropenic patients are susceptible to oropharyngeal candidiasis [36]. It is possible that the lack of IL-6 and G-CSF production by vaginal ECs may affect neutrophil function *in vivo* (once recruited by IL-8 and GM-CSF) resulting in detrimental rather than beneficial effects and an associated high fungal burden. Alternatively, the paucity of G-CSF and low IL-8 levels (compared with oral ECs), may suggest lower levels of neutrophil recruitment because they are detrimental vaginally or that vaginal ECs may not be as effective as oral ECs in regulating the neutrophil-mediated inflammatory response once initiated.

Of specific interest is CCL20, which recruits and activates CCR6-expressing dendritic cells, B cells and T cell subsets [37], [38]. The lack of CCL20 production by vaginal ECs is likely to reduce the rate of myeloid/lymphoid cell infiltration into vaginal tissues during *C. albicans* infection, resulting in poor activation of cellular immunity that is a typical feature of vaginal candidiasis [33]. Indeed, in a series of studies in a mouse model of vaginal candidiasis, although dendritic cells do infiltrate the vaginal mucosa, there is little or no evidence for dendritic cell activation or T cell infiltration which is central to activation of cellular immunity [33], [39]–[41]. More detailed investigations are required but the combined lack of IL-6, G-CSF and CCL20 secretion by vaginal ECs may contribute to the differential immune responses that are observed between oral and vaginal sites during *C. albicans* infection. In addition, the differences between oral and vaginal data sets may also be explained in part by the fact that vaginal ECs are at a reproductive site and may have evolved to be more tolerant to microbial pathogens and environmental stresses, thus inducing a weaker immune response or at least fewer cytokines. Irrespective, the combined features of *C. albicans* hypha formation/detection and differential cytokine profiles between oral and vaginal ECs may be the key processes that contribute to ‘immune compartmentalization’ at these mucosal sites and thereby host protection, unresponsiveness or susceptibility to superficial *C. albicans* infections.

## References

[1] Sobel JD (1988). Pathogenesis and epidemiology of vulvovaginal candidiasis.. Annals N Y Acad Sci.

[2] Moyes DL, Runglall M, Murciano C, Shen C, Nayar D (2010). A biphasic innate immune MAPK response discriminates between the yeast and hyphal forms of *Candida albicans* in epithelial cells.. Cell Host Microbe.

[3] Weindl G, Naglik JR, Kaesler S, Biedermann T, Hube B (2007). Human epithelial cells establish direct antifungal defense through TLR4-mediated signaling.. J Clin Invest.

[4] Fidel PL, Barousse M, Espinosa T, Ficarra M, Sturtevant J (2004). An intravaginal live *Candida* challenge in humans leads to new hypotheses for the immunopathogenesis of vulvovaginal candidiasis.. Infect Immun.

[5] Naglik JR, Moyes D, Makwana J, Kanzaria P, Tsichlaki E (2008). Quantitative expression of the *Candida albicans* secreted aspartyl proteinase gene family in human oral and vaginal candidiasis.. Microbiology.

[6] Gillum AM, Tsay EY, Kirsch DR (1984). Isolation of the *Candida albicans* gene for orotidine-5′-phosphate decarboxylase by complementation of *S. cerevisiae* ura3 and E. coli pyrF mutations.. Mol Gen Genet.

[7] Murad AM, Lee PR, Broadbent ID, Barelle CJ, Brown AJ (2000). CIp10, an efficient and convenient integrating vector for *Candida albicans*.. Yeast.

[8] Murad AM, Leng P, Straffon M, Wishart J, Macaskill S (2001). NRG1 represses yeast-hypha morphogenesis and hypha-specific gene expression in *Candida albicans*.. EMBO J.

[9] Lo HJ, Kohler JR, DiDomenico B, Loebenberg D, Cacciapuoti A (1997). Nonfilamentous *C. albicans* mutants are avirulent.. Cell.

[10] Schaller M, Zakikhany K, Naglik JR, Weindl G, Hube B (2006). Models of oral and vaginal candidiasis based on in vitro reconstituted human epithelia.. Nat Protoc.

[11] Liu Y, Shepherd EG, Nelin LD (2007). MAPK phosphatases - regulating the immune response.. Nat Rev Immunol.

[12] Brondello JM, Pouysségur J, McKenzie FR (1999). Reduced MAP kinase phosphatase-1 degradation after p42/p44 MAPK-dependent phosphorylation.. Science.

[13] Shaywitz AJ, Greenberg ME (1999). CREB: A stimulus-induced transcription factor activated by a diverse array of extracellular signals.. Ann Rev Biochem.

[14] Wisdom R (1999). AP-1: One switch for many signals.. Exper Cell Res.

[15] Schaller M, Mailhammer R, Grassl G, Sander CA, Hube B (2002). Infection of human oral epithelia with *Candida* species induces cytokine expression correlated to the degree of virulence.. J Invest Dermatol.

[16] Zakikhany K, Naglik JR, Schmidt-Westhausen A, Holland G, Schaller M (2007). In vivo transcript profiling of *Candida albicans* identifies a gene essential for interepithelial dissemination.. Cell Microbiol.

[17] Korting H, Hube B, Oberbauer S, Januschke E, Hamm G (2003). Reduced expression of the hyphal-independent *Candida albicans* proteinase genes *SAP1* and *SAP3* in the *efg1* mutant is associated with attenuated virulence during infection of oral epithelium.. J Med Microbiol.

[18] Fidel P, Luo W, Steele C, Chabain J, Baker M (1999). Analysis of vaginal cell populations during experimental vaginal candidiasis.. Infect Immun.

[19] Black CA, Eyers FM, Russell A, Dunkley ML, Clancy RL (1998). Acute neutropenia decreases inflammation associated with murine vaginal candidiasis but has no effect on the course of infection.. Infect Immun.

[20] Steele C, Ratterree M, Fidel PL (1999). Differential susceptibility of two species of macaques to experimental vaginal candidiasis.. J Infect Dis.

[21] Steele C, Ozenci H, Luo W, Scott M, Fidel PL (1999). Growth inhibition of *Candida albicans* by vaginal cells from naive mice.. Medical Mycol.

[22] Barousse MM, Steele C, Dunlap K, Espinosa T, Boikov D (2001). Growth inhibition of *Candida albicans* by human vaginal epithelial cells.. J Infect Dis.

[23] Nomanbhoy F, Steele C, Yano J, Fidel PL (2002). Vaginal and oral epithelial cell anti-*Candida* activity.. Infect Immun.

[24] Yano J, Lilly E, Barousse M, Fidel PL (2010). Epithelial cell-derived S100 calcium-binding proteins as key mediators in the hallmark acute neutrophil response during *Candida* vaginitis.. Infect Immun.

[25] Murciano C, Yanez A, Gil ML, Gozalbo D (2007). Both viable and killed *Candida albican*s cells induce in vitro production of TNF-alpha and IFN-gamma in murine cells through a TLR2-dependent signalling.. Eur Cytokine Netw.

[26] Netea MG, Gow NA, Munro CA, Bates S, Collins C (2006). Immune sensing of *Candida albicans* requires cooperative recognition of mannans and glucans by lectin and Toll-like receptors.. J Clin Invest.

[27] Romani L (2004). Immunity to fungal infections.. Nat Rev Immunol.

[28] Guma M, Stepniak D, Shaked H, Spehlmann ME, Shenouda S (2011). Constitutive intestinal NF κB does not trigger destructive inflammation unless accompanied by MAPK activation.. J Exp Med.

[29] Dongari-Bagtzoglou A, Kashleva H, Villar CC (2004). Bioactive interleukin-1alpha is cytolytically released from *Candida albicans*-infected oral epithelial cells.. Med Mycol.

[30] Dongari-Bagtzoglou A, Kashleva H (2003). Granulocyte-macrophage colony-stimulating factor responses of oral epithelial cells to *Candida albicans*.. Oral Microbiol Immunol.

[31] Sims JE, Smith DE (2010). The IL-1 family: regulators of immunity.. Nat Rev Immunol.

[32] Stylianou E, Saklatvala J (1998). Interleukin-1.. Int J Biochem Cell Biol.

[33] Fidel PL (2007). History and update on host defense against vaginal candidiasis.. Amer J Repro Immunol.

[34] Eversole LR, Reichart PA, Ficarra G, Schmidt-Westhausen A, Romagnoli P (1997). Oral keratinocyte immune responses in HIV-associated candidiasis.. Oral Surg Oral Med Oral Pathol Oral Radiol Endod.

[35] Schaller M, Boeld U, Oberbauer S, Hamm G, Hube B (2004). Polymorphonuclear leukocytes (PMNs) induce protective Th1-type cytokine epithelial responses in an in vitro model of oral candidosis.. Microbiology.

[36] Odds FC (1988). *Candida* and Candidosis.

[37] Le BM, Etchart N, Goubier A, Lira SA, Sirard JC (2006). Dendritic cells rapidly recruited into epithelial tissues via CCR6/CCL20 are responsible for CD8+ T cell crosspriming in vivo.. Immunity.

[38] Williams IR (2006). CCR6 and CCL20: partners in intestinal immunity and lymphorganogenesis.. Ann N Y Acad Sci.

[39] LeBlanc DM, Barousse MM, Fidel PL (2006). Role for dendritic cells in immunoregulation during experimental vaginal candidiasis.. Infect Immun.

[40] Fidel PLJ (2005). Immunity in vaginal candidiasis.. Curr Opin Infect Dis.

[41] Fidel PLJ (2004). History and new insights into host defense against vaginal candidiasis.. Trends Microbiol.

